# Consumer credit as a novel marker for economic burden and health after cancer in a diverse population of breast cancer survivors in the USA

**DOI:** 10.1007/s11764-017-0669-1

**Published:** 2018-01-25

**Authors:** Lorraine T. Dean, Kathryn H. Schmitz, Kevin D. Frick, Lauren H. Nicholas, Yuehan Zhang, S. V. Subramanian, Kala Visvanathan

**Affiliations:** 1 Department of Epidemiology, Johns Hopkins Bloomberg School of Public Health, Johns Hopkins University, 615 N Wolfe St, E6650, Baltimore, MD 21205 USA; 2 Department of Health Policy and Management, Johns Hopkins Bloomberg School of Public Health, Johns Hopkins University, Baltimore, MD USA; 3 Department of Oncology, Johns Hopkins School of Medicine, Baltimore, MD USA; 4 Department of Public Health Sciences, Pennsylvania State University College of Medicine, Pennsylvania State University, Hershey, PA USA; 5 Johns Hopkins Carey Business School, Johns Hopkins University, Baltimore, MD USA; 6 Department of General Internal Medicine, University of Pennsylvania School of Medicine, University of Pennsylvania, Philadelphia, PA USA; 7 Department of Social and Behavioral Sciences, Harvard T.H. Chan School of Public Health, Harvard University, Boston, MA USA

**Keywords:** Credit, Economic burden, Socioeconomic position, Breast cancer, Lymphedema, Survivorship, USA

## Abstract

**Background:**

Consumer credit may reflect financial hardship that patients face due to cancer treatment, which in turn may impact ability to manage health after cancer; however, credit’s relationship to economic burden and health after cancer has not been evaluated.

**Methods:**

From May to September 2015, 123 women with a history of breast cancer residing in Pennsylvania or New Jersey completed a cross-sectional survey of demographics, socioeconomic position, comorbidities, SF-12 self-rated health, economic burden since cancer diagnosis, psychosocial stress, and self-reported (poor to excellent) credit quality. Ordinal logistic regression evaluated credit’s contribution to economic burden and self-rated health.

**Results:**

Mean respondent age was 64 years. Mean year from diagnosis was 11.5. Forty percent of respondents were Black or Other and 60% were White. Twenty-four percent self-reported poor credit, and 76% reported good to excellent credit quality. In adjusted models, changing income, using savings, borrowing money, and being unable to purchase a health need since cancer were associated with poorer credit. Better credit was associated with 7.72 ([1.22, 14.20], *p* = 0.02) higher physical health *t*-score, and a − 2.00 ([− 3.92, − 0.09], *p* = 0.04) point change in psychosocial stress.

**Conclusions:**

This exploratory analysis establishes the premise for consumer credit as a marker of economic burden and health for breast cancer survivors. Future work should validate these findings in larger samples and for other health conditions.

**Implications for Cancer Survivors:**

Stabilizing and monitoring consumer credit may be a potential intervention point for mitigating economic burden after breast cancer.

## Introduction

In the USA, one’s credit quality may dictate access to additional financial resources that can be leveraged to improve health. Consumer credit quality may be especially important to those with a history of cancer, given the extensive body of literature on high out-of-pocket costs leading to economic burden after cancer [1–13]. Nearly half of cancer survivors experience financial distress [14] even among those who are insured [2, 3, 6, 7, 15]. Cancer survivors’ risk of bankruptcy is up to five times higher than those with no cancer history [15, 16]; among patients with a history of cancer, bankruptcy has been associated with a 79% greater risk of early mortality [17]. Out-of-pocket costs are even higher for those with adverse treatment effects due to cancer, such as breast cancer-related lymphedema [18–26], or comorbidities [2, 27]. Financial challenges due to cancer can delay the fulfillment of needs in other areas of life [4, 6, 28] and lead to greater physical and mental health challenges and lower quality of life [29–33]. Cancer survivors of low socioeconomic position (SEP) experience greatest economic burden due to cancer [2, 3, 27, 34–37], but commonly used SEP measures (income, wealth, education, social status) [15] may not fully capture the additional resources to navigate economic challenges that are available to those with better credit quality; however, no studies have linked consumer credit to economic burden after cancer or assessed how credit might relate to physical and mental health outcomes for cancer survivors.

While there is a large body of literature on the relationship between debt and physical and mental physical health [38, 39], credit and debt are distinct. In the USA, credit scores are derived quantitative measures of a person’s financial history and cumulative series of financial decisions, based on the use and timely payment schedules of loans, credit cards, and debts for an individual [40, 41]. Credit quality is based on one’s financial history and cumulative series of financial decisions [40, 41] and better reflects debt/asset management than the amount of debt itself. For example, those with high debt may still have good credit quality if the payback schedule is consistently followed, while those with even low debt can have poor credit quality if the debt is not paid back consistently. Further, credit quality reflects actions taken to mitigate the impact of debt, including bankruptcy, which can affect credit ratings for up to 10 years, regardless of the actual amount of debt.

Only a few studies have linked credit to individual or population-based health conditions [40–44], and no studies have linked credit to health after cancer. One ecological study found small but significant increases in city-wide influenza severity among those who fell behind on debt payments, which would influence credit quality [43]. If an economic shock [43, 45] due to an acute disease like the flu is associated with consumer credit, the shock due to management of a chronic disease and adverse events arising from it could have larger and sustained impacts. To assess how an economic shock due to chronic disease might be associated with credit quality, this exploratory analysis assessed the relationships between current self-reported consumer credit quality and (a) economic burden since cancer, and (b) current mental and physical health in a sample of women who had experienced the chronic disease of breast cancer.

## Materials and methods

**Fig. 1:**
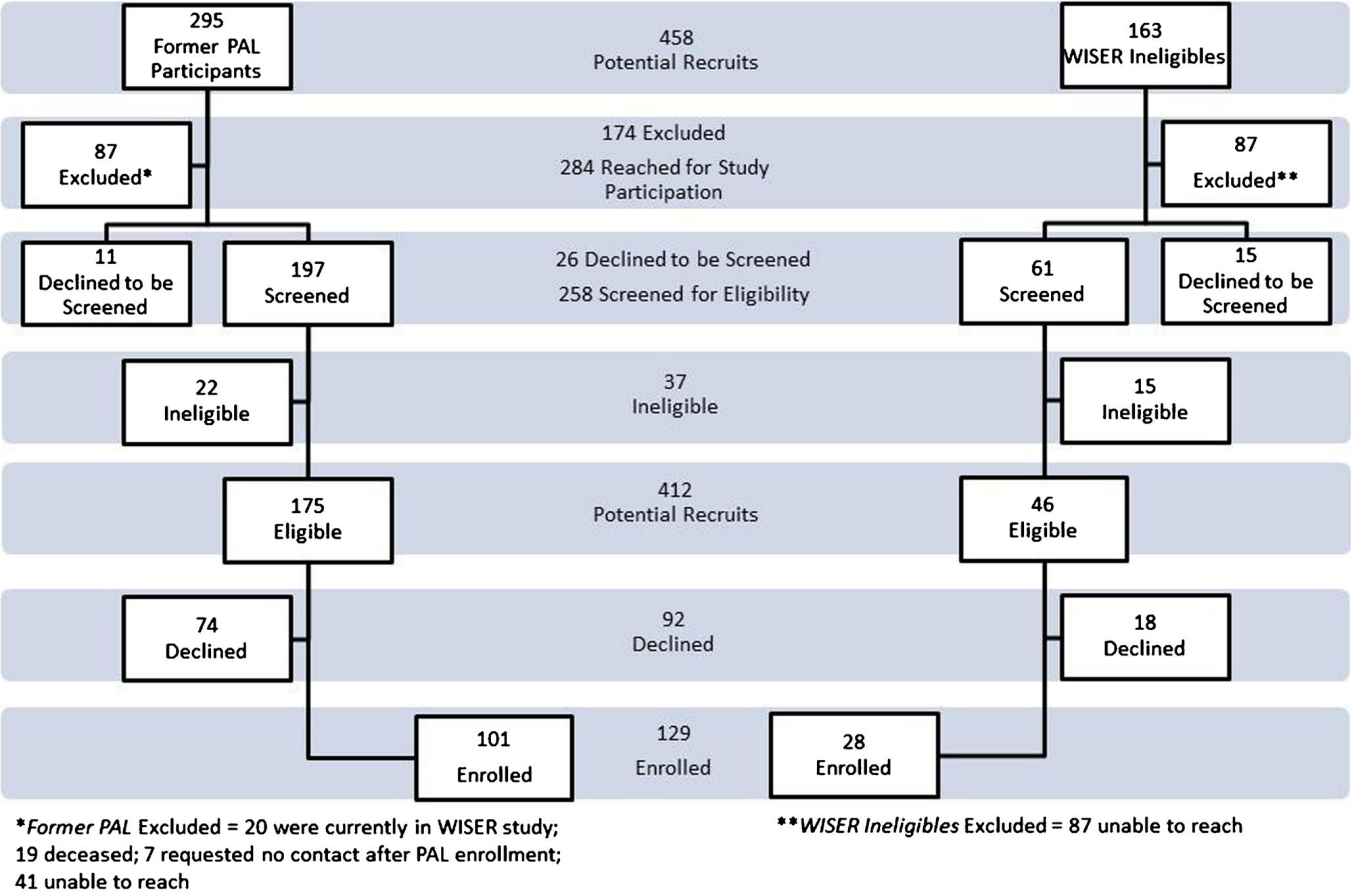
Recruitment consort diagram

Recruits were identified from prior participants of the Physical Activity and Lymphedema (PAL) trial (*n* = 295) [46, 47] or participants who were ineligible (*n* = 163) for the ongoing Women in Steady Exercise Research (WISER) Survivor Study (ClinicalTrials.gov #NCT01515124) [48] but met requirements for entry into PAL, who were still alive, had agreed to be contacted about future studies, and had updated contact information (Fig. 1). After these initial exclusions, 284 women were reached by phone in May to September of 2015 to be screened for eligibility in the PAL Social Economic and Quality of Life (PAL SEQL) follow-up study. Eligibility criteria included women with stage I–III invasive breast cancer after completion of active treatment, > 1 lymph node removed, and current resident of Pennsylvania or New Jersey. Those with active cancer or who were pregnant or planning to become pregnant in the next 6 months were excluded. Of the 284 reached, 26 declined prior to screening, leaving 258 who were screened for eligibility. Of those screened, 37 were ineligible, and 92 declined or dropped out due to not having time to participate in the study. Including those who declined before and after screening, the participation rate was 45%. Eligible non-participants were no different on any screening characteristic for which we could compare them to eligible participants. The study was approved by the Institutional Review Board of the University of Pennsylvania. Informed consent was obtained from all individual participants included in the study. After written consent, participants completed a baseline survey on credit, demographics, SEP, and health.

### Measures

#### Demographics and current socioeconomic position

Participants self-reported current age, and US census-defined race. SEP measures included education, total annual household income before taxes in 2014, number of people supported by that income, total summed value of financial assets (sum of checking, savings, stocks, and bonds for participant and partner), and self-rating of MacArthur ladder social status scale reflecting where the respondent perceives she stands at this time in her life relative to other persons in the USA [49]. Because reporting finances is sensitive and subject to high non-reporting [50], income and cash assets were collected as category measures rather than exact numbers.

#### Adjuvant cancer treatments and health history

Participants self-reported completing chemotherapy and/or radiation therapy and/or hormone therapy after cancer surgery, and year of breast cancer diagnosis. Self-report of breast cancer treatment has been validated as over 90% accurate [51]. The total number of types of adjuvant treatments was included as covariate, rather than each as separate treatment variables, to prevent model overfitting due to a large number of covariates for this sample size. Over 98% of participants had insurance in the past year; thus, insurance status was not included as a covariate due to lack of variation. Participants self-reported any of 23 comorbidities and previous diagnosis of breast cancer-related lymphedema, an adverse effect of cancer treatment.

*Perceptions of current credit quality* were self-reported as the response to the question “How would you rate your consumer credit?: Poor, Fair, Good, Very Good, Excellent, and I don’t know/I don’t have any consumer credit, which was further combined into two categories of Poor/Fair and Good/Very good/Excellent. Six responses of I don’t know/I don’t have any consumer credit were dropped. A subset of participants (*n* = 73) later retrieved and reported a quantitative credit scores obtained within the last year from a US credit-reporting company or personal credit card, which was used as a numeric continuous variable. The majority of the scores (77.0%) were obtained from the lendingtree.com, with the remainder from a credit bureau (21.7%) or personal credit card statement (1.4%).

*Economic burden since cancer* was assessed based on seven self-reported questions from the previously validated Breast Cancer Finances Survey [52, 53] on economic events occurring at any time since the cancer diagnosis: a change in income, and of those, a loss of income due to health; unemployment; increase in insurance premiums; using personal savings or retirement funds to cover health needs; borrowing money from others to cover a health need; or being unable to take care of health (“having an unmet health need”) due to limited finances.

#### Current health status

Physical and mental health status was assessed with composite and separate items from the 12-item Short Form Survey (SF-12) [54], with higher scores indicating better health.

#### Current psychosocial stress

The validated 10-item Perceived Stress Scale (PSS-10) assessed the degree to which a participant believed her life was uncontrollable or unpredictable in the past month [55]. Higher scores indicate more perceived stress.

### Statistical analysis

Means and percentages of participant characteristics by credit quality were compared using χ^2^ tests or Fisher’s exact tests for variables with categories that had < 5 responses, and ANOVA. Associations between credit quality and dichotomous outcomes for economic burden since cancer diagnosis were evaluated using logistic regression. The economic burden scale items for loss of income and unemployment were dropped due to < 20 respondents endorsing them. Continuous outcomes of SF-12 health *t*-scores and perceived stress scores were evaluated using linear regression. All models adjusted for age based on previous literature suggesting better credit with age [41], and number of cancer treatment modalities, and years since cancer diagnosis. Lymphedema and comorbidities were included as confounders that are known to be a source of economic burden for those with a cancer history [2, 18–27]. Additionally, models adjusted for race and SEP variables, due to previous research suggesting that credit-scoring models penalize borrowers for using the types of credit that are disproportionately marketed to, and thus used by, racial/ethnic minorities [53], and those who live in economically disadvantaged communities [54]. All *p* values are two-sided.

## Results

Descriptive statistics in Table 1 are based on an analytic sample of the 123 participants who responded to the self-reported credit quality question of the total 129 participants enrolled in the study. Mean age was 64 years, and 40% of the sample was Black or some Other race, and 60% White. Approximately 24% had poor credit quality, and 76% reported good to excellent credit quality, which reflected subsequently higher objective credit scores. Among the 73 participants providing numeric scores, subjective credit quality and objective numeric score were highly correlated (0.81). Most participants had some college (76%), and annual household income greater than $70,000 (44%), supporting an average of two people including the participant. A little more than half had assets of < $50,000, and participants, on average, rated themselves as mid-level in social status. Mean time since cancer diagnosis was 11.5 years, and participants had an average of two cancer treatment modalities. Roughly half had lymphedema, and a little more than one third had two or more comorbidities. Over half reported experiencing a change in income since their cancer diagnosis (58%). Fewer than half experienced increases in insurance premiums (46%) or had to use their savings to cover expenses (38%), while less than one fifth borrowed money to pay for a health need (17%), or were unable to purchase a health need (19%) since their cancer diagnosis. Current composite physical health (46.3) was within the range of normative values for healthy females in the USA 45 to 75+ (ranges from 39 to 50), while composite mental health (50.5) was average (ranges 50–52) [56]. Mean perceived stress scores (19.8) were higher than population-based averages (16.1) for women of the same age range [57].

**Table 1:** Characteristics of study population (*N* = 123)

	Mean (SD) or *N* (%) [range]	Self-reported credit quality mean (SD) or *N* (%) [range]		*p* ^a^
		Poor/Fair *n*=30 (24.4%)	Good to Excellent *n*=93 (75.6%)	
DEMOGRAPHICS				
Age	64 (8) [45–88]	63(8)	64 (8)	0.56
Race				0.40
Black and Other	49 (39.8%)	10 (33.3%)	39 (41.9%)	
White	74 (60.2%)	20 (66.7%)	54 (58.1%)	
Current consumer credit				
Credit score, mean (*n* = 73)	742 (96) [465–843]	577 (76) [465–761]	778 (52) [648–829]	< 0.001
CURRENT SOCIOECONOMIC POSITION				
Education				0.93
≤ High school degree	29 (24.4%)	7 (25%)	22 (24.2%)	
Some college or more	90 (75.6%)	21 (75%)	69 (75.8%)	
Household income (*n* = 118)				< 0.001
≤ $30,000	18 (15.3%)	11 (37.9%)	7 (7.9%)	
$30,001–$70,000	48 (40.7%)	14 (48.3%)	34 (38.2%)	
> $70,000	52 (44.1%)	4 (13.8%)	48 (53.9%)	
Number supported by income	2 (1) [1–7]	2 (1) [1–3]	2 (1) [1–7]	0.01
Total cash assets (*n* = 113)				< 0.001
≤ 49,999	60 (53.1%)	25 (83.3%)	35 (42.2%)	
≥ $50,000	53 (46.9%)	5 (16.7%)	48 (57.8%)	
Social status	6.2 (1.8) [1–10]	4.5 (1.6) [1–8]	6.8 (1.6) [3–10]	< 0.001
CANCER TREATMENT AND HEALTH HISTORY				
Years since diagnosis (*n* = 122)	11.5 (4.5) [0–23]	12 (4.2) [5–20]	11 (4.6) [0–23]	0.21
Number of adjuvant cancer treatment modalities (*n* = 120)	2 (1) [0–3]	2 (1) [1–3]	2 (1) [0–3]	0.40
Lymphedema				
No	66 (53.7%)	12 (40.0%)	54 (58.1%)	0.08
Yes	57 (46.3%)	18 (60.0%)	39 (41.9%)	
Comorbid conditions				0.15
0 or 1	79 (64.2%)	16 (20.3%)	63 (67.7%)	
2+	44 (35.8%)	14 (31.8%)	30 (32.3%)	
ECONOMIC BURDEN OUTCOMES SINCE CANCER				
Change in income (*n* = 118)	68 (57.6%)	22 (75.9%)	46 (51.7%)	0.02
Insurance premiums increased (*n* = 121)	56 (46.3%)	17 (58.6%)	39 (42.4%)	0.13
Used savings (*n* = 122)	46 (37.7%)	21 (72.4%)	25 (26.9%)	< 0.001
Borrowed money (*n* = 121)	21 (17.4%)	13 (44.8%)	8 (8.8%)	< 0.001
Unable to purchase health need (*n* = 119)	23 (19.3%)	15 (53.6%)	8 (8.8%)	< 0.001
CURRENT HEALTH OUTCOMES				
SF-12 health (*t*-scores)				
Composite physical health	46.2 (11.6) [13.7–65.5]	35.8 (13.1) [17.0–60.0]	48.9 (9.8) [13.7–65.5]	< 0.001
General health	43.7 (10.5) [26.6–63.8]	36.2 (7.9) [26.6–51.4]	45. 8 (10.3) [26.6–63.8]	< 0.001
Physical functioning	46.6 (13.5) [13.1–56.8]	37.3 (17.0) [13.1–56.8]	49.4 (11.2) [13.1–56.8]	< 0.001
Role limitations—physical^b^	47.1 (8.3) [26.0–55.6]	42.3 (8.8) [26.0–55.6]	41.5 (8.4) [33.4–55.6]	< 0.001
Bodily pain	50.0 (11.8) [18.0–60.4]	44.3 (14.4) [18.0–60.4]	52.2 (9.7) [28.6–60.4]	0.001
Composite mental health	50.5 (9.1) [14.8–68.8]	47.1 (8.3) [33.3–60.0]	51.3 (9.1) [14.8–68.8]	0.05
Vitality	48.2 (11.2) [20.7–68.7]	41.6 (11.8) [20.7–56.7]	50.0 (10.3) [20.7–68.7]	0.004
Social functioning	48.7 (10.9) [23.8–57.3]	41.6 (11.3) [23.8–57.3]	51.2 (9.5) [23.8–57.3]	< 0.001
Role limitations—emotional^b^	50.5 (7.0) [25.4–55.7]	46.3 (8.5) [25.4–55.7]	51.8 (5.8) [33.0–55.7]	0.002
Mental health	48.4 (10.6) [15.4–64.0]	42.9 (9.5) [29.3–57.0]	49.8 (10.4) [15.4–64.0]	0.002
Perceived stress (PSS-10)^c^	19.8 (3.4) [11–28]	21.3 (3.8) [14–28]	19.3 (3.0) [11–26]	0.004

^a^Associations between credit quality and key covariates were evaluated using contingency tables. All *P*-values are two-sided

^b^Higher SF-12 scores correspond to more favorable health outcomes

^c^Higher PSS-10 scores correspond to higher stress

Participants with higher household income, more people supported by that income, more cash assets, and higher social status had significantly better credit quality than those of lower SEP (*p* < 0.01). There was no difference in self-reported credit quality based on number of years since cancer diagnosis, cancer treatment modalities, presence of breast cancer-related lymphedema, or comorbidities. Those with better credit quality were significantly less likely to have had a change in income (*p* = 0.02), used savings, borrowed money, or had been unable to purchase a health need since cancer (*p* < 0.001). Correlations among economic burden events ranged from − 0.18 to 0.52. Means for all SF-12 *t*-scores were significantly higher for participants with better credit quality (*p* < 0.05), while lower stress scores were found in better self-reported credit categories (*p* = 0.004).

Tables 2 and 3 present results on the relationship between self-reported credit quality and economically burdensome events (Table 2) and health outcomes (Table 3). After adjusting for race, age, SEP, cancer treatments, years since diagnosis, lymphedema, and comorbid conditions, log odds of having a change in income (− 1.52 [− 2.93, − 0.11], *p* = 0.03), using savings (− 1.42 [− 2.73, − 0.11], *p* = 0.03), borrowing money (− 2.51 [− 4.20, − 0.82], *p* = 0.004), and being unable to purchase a health need (− 2.07 [− 3.62, − 0.52], *p* = 0.009), all since cancer, were associated with poorer consumer credit. SF-12 composite physical health *t*-score (7.72 [1.22, 14.20], *p* = 0.02) was the most strongly associated with credit quality, followed by vitality (7.80 [1.76, 13.85], *p* = 0.01), general health (7.03 [1.55, 12.50], *p* = 0.01), and social functioning (6.62 [1.11, 12.12], *p* = 0.02). Better credit was significantly associated with a lower perceived stress score (− 2.00 [− 3.92, − 0.09], *p* = 0.04).

**Table 2:** Log odds (logit regressions) of credit quality and economic burden since breast cancer

	Self-reported credit quality^a^		
	*β* (SE)	95% CI	*p*
Change in income since cancer	− 1.52 (0.72)	− 2.93, − 0.11	0.03
Insurance premiums increased since cancer	− 0.66 (0.64)	− 1.92, 0.39	0.30
Used savings since cancer	− 1.42 (0.67)	− 2.73, − 0.11	0.03
Borrowed money since cancer	− 2.51 (0.86)	− 4.20, − 0.82	0.004
Unable to purchase health need since cancer	− 2.07 (0.79)	− 3.62, − 0.52	0.009

**Table 3:** Linear coefficients of credit quality and health after breast cancer

	Outcome: self-reported credit quality^a^		
	β (SE)	95% CI	*p*
Composite physical health	7.72 (3.26)	1.22, 14.20	0.02
General health	7.03 (2.75)	1.55, 12.50	0.01
Physical function	3.48 (3.77)	− 4.01, 10.97	0.36
Role limitations due to physical function^b^	4.63 (2.38)	− 0.10, 9.36	0.06
Bodily pain	4.83 (3.25)	− 1.63, 11.30	0.14
Composite mental health	2.96 (2.85)	− 2.71, 8.63	0.30
Vitality	7.80 (3.01)	1.76, 13.85	0.01
Social functioning	6.62 (2.77)	1.11, 12.12	0.02
Mental health	5.13 (3.09)	− 1.02, 11.27	0.10
Role limitations due to emotional function^**b**^	2.39 (1.98)	− 1.54, 6.32	0.23
Perceived stress (PSS-10)^**b**^	− 2.00 (0.96)	− 3.92, − 0.09	0.04

^a^Adjusted for age, race, SEP (education, income, number being supported by that income, assets, social status), number of cancer treatments, years since diagnosis, presence of lymphedema, and comorbid conditions

^b^Higher SF-12 *t*-scores correspond to more favorable health outcomes. Higher PSS-10 scores correspond to higher stress

## Discussion

Building upon previous studies linking self-reported credit quality to health [40–44], this exploratory study assessed whether or not self-reported consumer credit, net of socioeconomic factors, and comorbid conditions were associated with economic burden and mental and physical health after cancer, among a sample of long-term breast cancer survivors. Results showed that some domains of economic burden after cancer including having a change in income, using savings, borrowing money, and being unable to purchase a health need were each associated with poorer self-reported credit. Overall physical and general health, mental health subdomains of vitality and social functioning, and less psychosocial stress were associated with better self-reported credit.

Consumer credit quality may reflect access to additional forms of capital, like savings accounts or financial help from other sources that can be leveraged to buffer economic burden after chronic disease. This is especially important for cancer survivors, who are more likely than those with no cancer history to experience job changes, have fewer financial resources, or experience increased insurance costs [58–60]. The results of this exploratory analysis provided partial support of this pathway; however, the study was not powered to assess the impact of income loss or unemployment specifically. Additionally, results suggest that using savings, borrowing money, and being unable to purchase a health need might be financial distress signals that are reflected in consumer credit ratings. Those who use up their personal savings may leverage credit to take out personal loans to cover medical care or medical debt. Having greater debt itself may lead to poorer credit quality, but credit in any amount that is not well managed or results in defaults could lead to detrimental hits to credit quality. The need to leverage credit itself may be a sign of financial distress that reflects underlying unmet needs after cancer, which has been associated with lower mental quality of life [29, 61] and greater emotional distress among cancer survivors [30].

Better self-reported consumer credit quality was associated with better physical health, vitality, better social functioning, and less stress, which may suggest that having good credit opens access to resources to buffer how much poor physical health interferes with day-to-day activities and social affairs. For example, the need for vitality to manage affairs may be especially important for those with a history of cancer, who may suffer from cancer-related fatigue that limits their ability to handle daily affairs [62], like managing credit, and conducting social affairs. The present results provide partial support of a relationship between credit and mental health outcomes of vitality and social functioning. Worse consumer credit may be associated with psychosocial stress by reflecting the context of economic hardship that increases allostatic load [63]; however, a relationship with allostatic load would need to be explored empirically in other studies.

While promising, using consumer credit in health studies faces challenges of validity and generalizability, which have contributed to this study’s limitations. Self-reported credit is not a validated measure but in this sample was highly correlated (*r* = 0.81) with the objective quantitative scores suggesting that self-reported credit quality may be a strong substitute for credit score. Cognitive interviewing for this item may have improved its validity. Credit scores retrieved from different sources may not be equivalent in what they represent or be calculated from equivalent information: a score of 700 may be good by one model’s standards, but fair by another. Thus, self-reported credit quality may be a better measure than numeric scores for assessing self-reported health outcomes, because it captures the perceived influence that consumer credit has on everyday life.

As a cross-sectional study with a one-time measure of credit, the direction of association between credit and health could not be assessed. Thus, it is possible that those who have managed poor health for many years were less likely to maintain income and earnings, which led to poor credit. Longitudinal analysis of credit and health outcomes could illuminate this pathway, and this exploratory study establishes the premise of an association that should be further explored. Given a 45% study participation rate, the results may not reflect what would have been found in a sample that had a larger proportion of eligible participants joining the study; yet, 45% is a participation rate that is in line with well-respected population-based surveys such as the Behavioral Risk Factor Surveillance Survey (BRFSS) [64]. The results may not be generalizable beyond this sample of women with a history of breast cancer or beyond the US context for consumer credit; however, this exploratory analysis sets a precedent for these associations to be explored in broader samples.

## Conclusions

In conclusion, consumer credit quality may reflect both economically burdensome events and health outcomes after cancer and could be a useful inclusion in screening for financial distress [65]. While future studies will be necessary to elucidate the extent to which consumer credit can predict health outcomes in the broader population, this exploratory analysis sets a foundation for pathways to explore. Future work should explore associations between credit and additional cancer-related health outcomes across broader populations and geographies and use longitudinal data to assess the causal pathways that might link credit and health.

## Implications for cancer survivors

Altogether, results of this exploratory study suggest that consumer credit shows promise as a unique contribution to understanding the long-term financial impact of breast cancer, which may have implications for health after cancer. If these results are validated in larger studies, implications for cancer survivors include encouraging policies to stabilize consumer credit that arises due to medical debt, or monitoring changes in consumer credit for cancer survivors to determine when intervention is necessary to prevent bankruptcy or long-term economic strain.

## References

[1] Jagsi R, Pottow JA, Griffith KA, Bradley C, Hamilton AS, Graff J (2014). Long-term financial burden of breast cancer: experiences of a diverse cohort of survivors identified through population-based registries. J Clin Oncol.

[2] Bernard DS, Farr SL, Fang Z (2011). National estimates of out-of-pocket health care expenditure burdens among nonelderly adults with cancer: 2001 to 2008. J Clin Oncol.

[3] Guy GP, Yabroff KR, Ekwueme DU, Virgo KS, Han X, Banegas MP (2015). Healthcare expenditure burden among non-elderly cancer survivors, 2008–2012. Am J Prev Med.

[4] Zafar SY (2016). Financial toxicity of cancer care: it’s time to intervene. J Natl Cancer Inst.

[5] Zafar SY, Abernethy AP (2013). Financial toxicity, part I: a new name for a growing problem. Oncology (Williston Park).

[6] Zafar SY, Peppercorn JM, Schrag D, Taylor DH, Goetzinger AM, Zhong X, et al. The financial toxicity of cancer treatment: a pilot study assessing out-of-pocket expenses and the insured cancer patient's experience. Oncologist. 2013;18(4):381–90. 10.1634/theoncologist.2012-0279. 10.1634/theoncologist.2012-0279 PMC3639525 23442307

[7] Arozullah AM, Calhoun EA, Wolf M, Finley D, Fitzner KA, Heckinger EA (2004). The financial burden of cancer: estimates from a study of insured women with breast cancer. J Support Oncol.

[8] Pisu M, Azuero A, McNees P, Burkhardt J, Benz R, Meneses K (2010). The out of pocket cost of breast cancer survivors: a review. J Cancer Surviv.

[9] Finkelstein EA, Tangka FK, Trogdon JG, Sabatino SA, Richardson LC. The personal financial burden of cancer for the working-aged population. 2009. 19895184

[10] Latremouille-Viau D, Chang J, Guerin A, Shi S, Wang E, Yu J, et al. The economic burden of common adverse events associated with metastatic colorectal cancer treatment in the United States. J Med Econ. 2017;20(1):54–62. 10.1080/13696998.2016.1225577. 10.1080/13696998.2016.1225577 27603498

[11] Narang AK, Nicholas LH. Out-of-pocket spending and financial burden among medicare beneficiaries with cancer. JAMA Oncology. 2016; 10.1001/jamaoncol.2016.4865 PMC5441971 27893028

[12] Barron JJ, Quimbo R, Nikam PT, Amonkar MM (2008). Assessing the economic burden of breast cancer in a US managed care population. Breast Cancer Res Treat.

[13] Pisu M, Azuero A, Benz R, McNees P, Meneses K (2017). Out-of-pocket costs and burden among rural breast cancer survivors. Cancer Med.

[14] Altice CK, Banegas MP, Tucker-Seeley RD, Yabroff KR (2017). Financial hardships experienced by cancer survivors: a systematic review. J Natl Cancer Inst.

[15] Banegas MP, Guy GP, de Moor JS, Ekwueme DU, Virgo KS, Kent EE, et al. For working-age cancer survivors, medical debt and bankruptcy create financial hardships. Health Aff. 2016;35(1):54–61. 10.1377/hlthaff.2015.0830. 10.1377/hlthaff.2015.0830 PMC6057727 26733701

[16] Ramsey S, Blough D, Kirchhoff A, Kreizenbeck K, Fedorenko C, Snell K, et al. Washington State cancer patients found to be at greater risk for bankruptcy than people without a cancer diagnosis. Health Aff. 2013;32(6):1143–52. 10.1377/hlthaff.2012.1263. 10.1377/hlthaff.2012.1263 PMC4240626 23676531

[17] Ramsey SD, Bansal A, Fedorenko CR, Blough DK, Overstreet KA, Shankaran V, et al. Financial insolvency as a risk factor for early mortality among patients with cancer. J Clin Oncol. 2016;34(9):980–6. 10.1200/JCO.2015.64.6620. 10.1200/JCO.2015.64.6620 PMC4933128 26811521

[18] Shih Y-CT XY, Cormier JN, Giordano S, Ridner SH, Buchholz TA (2009). Incidence, treatment costs, and complications of lymphedema after breast cancer among women of working age: a 2-year follow-up study. J Clin Oncol.

[19] Bennett CL, Calhoun EA (2007). Evaluating the total costs of chemotherapy-induced febrile neutropenia: results from a pilot study with community oncology cancer patients. Oncologist.

[20] Schmitz KH, DiSipio T, Gordon LG, Hayes SC (2015). Adverse breast cancer treatment effects: the economic case for making rehabilitative programs standard of care. Support Care Cancer.

[21] Schnur JB, Zivin JG, Mattson DM, Green S, Jandorf LH, Wernicke AG (2012). Acute skin toxicity-related, out-of-pocket expenses in patients with breast cancer treated with external beam radiotherapy. Support Care Cancer.

[22] Bilir SP et al. Economic burden of toxicities associated with treating metastatic melanoma in the United States. Am Health Drug Benefits. 2016;9(4):203–13. PMC5004818 27688833

[23] Irwin DE, Masaquel A, Johnston S, Barnett B (2016). Adverse event-related costs for systemic metastatic breast cancer treatment among female Medicaid beneficiaries. J Med Econ.

[24] Hansen RN, Ramsey SD, Lalla D, Masaquel A, Kamath T, Brammer M, et al. Identification and cost of adverse events in metastatic breast cancer in taxane and capecitabine based regimens. Spring. 2014;3(1):259. 10.1186/2193-1801-3-259. 10.1186/2193-1801-3-259 PMC4047276 24926422

[25] Hurvitz S, Guerin A, Brammer M, Guardino E, Zhou Z-Y, Viau DL (2014). Investigation of adverse-event-related costs for patients with metastatic breast cancer in a real-world setting. Oncologist.

[26] Rashid N, Koh HA, Baca HC, Lin KJ, Malecha SE, Masaquel A. Economic burden related to chemotherapy-related adverse events in patients with metastatic breast cancer in an integrated health care system. Breast Cancer Targets Ther. 2016;8:173. 10.2147/BCTT.S105618. 10.2147/BCTT.S105618 PMC5063632 27785099

[27] Davidoff AJ, Erten M, Shaffer T, Shoemaker JS, Zuckerman IH, Pandya N, et al. Out-of-pocket health care expenditure burden for Medicare beneficiaries with cancer. Cancer. 2013;119(6):1257–65. 10.1002/cncr.27848. 10.1002/cncr.27848 23225522

[28] Tucker-Seeley RD, Yabroff KR (2016). Minimizing the “financial toxicity” associated with cancer care: advancing the research agenda. J Natl Cancer Inst.

[29] Cheng K, Wong W, Koh C (2016). Unmet needs mediate the relationship between symptoms and quality of life in breast cancer survivors. Support Care Cancer.

[30] Meeker CR, Geynisman DM, Egleston BL, Hall MJ, Mechanic KY, Bilusic M, et al. Relationships among financial distress, emotional distress, and overall distress in insured patients with cancer. J Oncol Pract. 2016:JOPR011049;12(7):e755–64. 10.1200/JOP.2016.011049. 10.1200/JOP.2016.011049 PMC5015446 27328795

[31] de Souza JA, Yap BJ, Wroblewski K, Blinder V, Araujo FS, Hlubocky FJ (2016). Measuring financial toxicity as a clinically relevant patient-reported outcome: the validation of the COmprehensive Score for financial Toxicity. Cancer.

[32] de Jong M, Tamminga SJ, Frings-Dresen MHW, de Boer AGEM (2017). Quality of Working Life of cancer survivors: associations with health- and work-related variables. Support Care Cancer.

[33] Kale HP, Carroll NV (2016). Self-reported financial burden of cancer care and its effect on physical and mental health-related quality of life among US cancer survivors. Cancer.

[34] Souza JA, Yap BJ, Hlubocky FJ, Wroblewski K, Ratain MJ, Cella D (2014). The development of a financial toxicity patient-reported outcome in cancer: the COST measure. Cancer.

[35] Pisu M, Azuero A, Meneses K, Burkhardt J, McNees P (2011). Out of pocket cost comparison between Caucasian and minority breast cancer survivors in the Breast Cancer Education Intervention (BCEI). Breast Cancer Res Treat.

[36] Timmons A, Gooberman-Hill R, Sharp L (2013). “It’s at a time in your life when you are most vulnerable”: a qualitative exploration of the financial impact of a cancer diagnosis and implications for financial protection in health. PLoS One.

[37] Oatis W, Nonzee N, Markossian T, Shankaran V, McKoy J, Evens A, et al. Interpreting out-of-pocket expenditures for cancer patients: the importance of considering baseline household income information. J Clin Oncol. 2009;27(15S):6541. 10.1200/jco.2009.27.15s.6541

[38] Richardson T, Elliott P, Roberts R (2013). The relationship between personal unsecured debt and mental and physical health: a systematic review and meta-analysis. Clin Psychol Rev.

[39] Turunen E, Hiilamo H (2014). Health effects of indebtedness: a systematic review. BMC Public Health.

[40] Israel S, Caspi A, Belsky DW, Harrington H, Hogan S, Houts R (2014). Credit scores, cardiovascular disease risk, and human capital. Proc Natl Acad Sci U S A.

[41] Li Y, Gao J, Enkavi AZ, Zaval L, Weber EU, Johnson EJ (2015). Sound credit scores and financial decisions despite cognitive aging. Proc Natl Acad Sci.

[42] Brockett PL, Golden LL (2007). Biological and psychobehavioral correlates of credit scores and automobile insurance losses: toward an explication of why credit scoring works. J Risk Insur.

[43] Houle JN, Collins JM, Schmeiser MD (2015). Flu and finances: influenza outbreaks and loan defaults in US cities, 2004–2012. Am J Public Health.

[44] Lee Y, Song I (2015). A study on the economic factor associated with suicide: debt and suicide ideation. Mental Health Soc Work.

[45] Stegman MA, Quercia RG, Ratcliffe J, Ding L, Davis WR (2007). Preventive servicing is good for business and affordable homeownership policy. Housing Policy Debate.

[46] Schmitz KH, Ahmed RL, Troxel AB, Cheville A, Lewis-Grant L, Smith R, et al. Weight lifting for women at risk for breast cancer–related lymphedema: a randomized trial. JAMA. 2010;304(24):2699–705. 10.1001/jama.2010.1837. 10.1001/jama.2010.1837 21148134

[47] Schmitz KH, Troxel AB, Cheville A, Grant LL, Bryan CJ, Gross CR, et al. Physical Activity and Lymphedema (the PAL trial): assessing the safety of progressive strength training in breast cancer survivors. Contemp Clin Trials. 2009;30(3):233–45. 10.1016/j.cct.2009.01.001. 10.1016/j.cct.2009.01.001 PMC2730488 19171204

[48] Patterson RE, Colditz GA, Hu FB, Schmitz KH, Ahima RS, Brownson RC, et al. The 2011–2016 Transdisciplinary Research on Energetics and Cancer (TREC) initiative: rationale and design. Cancer Causes Control. 2013;24(4):695–704. 10.1007/s10552-013-0150-z. 10.1007/s10552-013-0150-z PMC3602225 23378138

[49] Adler N, Singh-Manoux A, Schwartz J, Stewart J, Matthews K, Marmot MG (2008). Social status and health: a comparison of British civil servants in Whitehall-II with European-and African-Americans in CARDIA. Soc Sci Med.

[50] Berkman L, Macintyre S. The measurement of social class in health studies: old measures and new formulations. IARC Sci Publ. 1997;138(51) 9353663

[51] Oberst K, Bradley CJ, Schenk M (2008). Breast and prostate cancer patient’s reliability of treatment reporting. J Registry Manag.

[52] Gordon L, Scuffham P, Hayes S, Newman B (2007). Exploring the economic impact of breast cancers during the 18 months following diagnosis. Psycho Onocol.

[53] Given BA, Given CW, Stommel M (1994). Family and out of pocket costs for women with breast cancer. Cancer Pract.

[54] Ware JE, Kosinski M, Keller SD (1996). A 12-Item Short-Form Health Survey: construction of scales and preliminary tests of reliability and validity. Med Care.

[55] Cohen S, Williamson G, Spacapam S, Oskamp S (1988). Perceived stress in a probability sample of the US. The social psychology of health: Claremont symposium on applied social psychology.

[56] Ware JE KM, Keller SD. SF-12: how to score the SF-12 Physical and Mental Health Summary Scales, Second Edition. Boston, MA 1995.

[57] Cohen S, JANICKI-DEVERTS D (2012). Who’s stressed? Distributions of psychological stress in the United States in probability samples from 1983, 2006, and 20091. J Appl Soc Psychol.

[58] Darby K, Davis C, Likes W, Bell J (2009). Exploring the financial impact of breast cancer for African American medically underserved women: a qualitative study. J Health Care Poor Underserved.

[59] De Boer AG, Taskila T, Ojajärvi A, van Dijk FJ, Verbeek JH (2009). Cancer survivors and unemployment: a meta-analysis and meta-regression. JAMA.

[60] Yin W, Horblyuk R, Perkins JJ, Sison S, Smith G, Snider JT, et al. Association between breast cancer disease progression and workplace productivity in the United States. J Occup Environ Med. 2017;59(2):198–204. 10.1097/JOM.0000000000000936. 10.1097/JOM.0000000000000936 28166126

[61] Hasegawa T, Goto N, Matsumoto N, Sasaki Y, Ishiguro T, Kuzuya N, et al. Prevalence of unmet needs and correlated factors in advanced-stage cancer patients receiving rehabilitation. Support Care Cancer. 2016;24(11):4761–7. 10.1007/s00520-016-3327-7. 10.1007/s00520-016-3327-7 27344328

[62] Bower JEG, Patricia A. Symptoms: fatigue and cognitive function. In: Ganz PA, editor. Improving outcomes for breast cancer survivors. Springer; 2015. p. 53–76.

[63] Solís CB, Fantin R, Castagné R, Lang T, Delpierre C, Kelly-Irving M (2016). Mediating pathways between parental socio-economic position and allostatic load in mid-life: findings from the 1958 British birth cohort. Soc Sci Med.

[64] Centers for Disease Control and Prevention. Behavioral Risk Factor Surveillance System 2016 Summary Data Quality Report2017 29 June 2017.

[65] Khera N, Holland JC, Griffin JM. Setting the stage for universal financial distress screening in routine cancer care. Cancer. 2017;123(21):4092–6. 10.1002/cncr.30940. 10.1002/cncr.30940 28817185

